# Link between the Platelet-to-Lymphocyte Ratio and Coronary Artery Disease Severity 

**Published:** 2016-04-13

**Authors:** Hilman Zulkifli Amin, Lukman Zulkifli Amin, Firman Zulkifli Amin

**Affiliations:** 1 *Faculty of Medicine, Universitas Indonesia, Jl. Salemba Raya No. 6, Jakarta, Indonesia. Tel: +62 81384967494. Fax: +62 213160493.E-mail: hilman_amin@yahoo.co.id.*; 2 *Department of Internal Medicine, Faculty of Medicine, Universitas Indonesia. Jl. Salemba Raya No. 6, Jakarta, Indonesia. Tel: +62 81384967494. Fax: +62 213160493.E-mail: lukman.zulkifli@gmaill.com.*; 3 *Faculty of Medicine, Universitas Indonesia, Jl. Salemba Raya No. 6, Jakarta, Indonesia. Tel: +62 81384967494. Fax: +62 213160493.E-mail: firman_za@hotmail.com.*

Coronary artery disease (CAD) is still the leading cause of morbidity and mortality around the world. Many risk factors such as hypertension, diabetes mellitus, and physical inactivity contribute to the progression of CAD. Complex inflammation processes have a role in initiating and developing atherosclerosis. Recently, the platelet-to-lymphocyte ratio (PLR) has been researched as a predictor of CAD severity.^1^^, ^^2^ Easily required and relatively inexpensive, this efficient modality could help to predict the severity of CAD, in addition to conventional risk factors and commonly used biomarkers such as C-reactive protein (CRP).^1^

Low blood lymphocytes and high circulating platelet counts separately have been known to correlate with worse cardiovascular consequences in patients with CAD.^3^^,^^4^ However, the PLR confers a novel idea that combines the risk prediction of these 2 parameters into 1. Additionally, it shows a new perspective on both aggregation and inflammation pathways; thus, it may be more valuable than either platelet or lymphocyte count alone as a severity predictor in CAD. The study by Yuksel et al.^1^ found that a high PLR level was independently associated with the severity of CAD. Patients with a high pre-procedural PLR showed a positive association with the Gensini score, one of the scoring methods in assessing CAD severity, inasmuch as they had a significantly higher Gensini score. The Gensini score categorizes the degree and extent of the coronary artery stenosis. The scoring is 1 point for 1% to 25% stenosis, 2 points for 26% to 50%, 4 points for 51% to 75%, 8 points for 76% to 90%, 16 points for 91% to 99%, and 32 points for total occlusion. Then, it is multiplied by a parameter to imply the significance of the lesion’s location in the coronary artery. It is multiplied by 5 for a left main lesion; 2.5 for the proximal left anterior descending (LAD) or left circumflex (LCX) artery; 1.5 for the mid-segment LAD and LCX; 1 for the distal segment of the LAD an LCX, first diagonal branch, first obtuse marginal branch, right coronary artery, posterior descending artery, and intermediate artery; and 0.5 for the second diagonal and obtuse marginal branches. This study also showed a positive correlation between the PLR and the CRP level; it may, therefore, suggest an inflammatory state. This recent study showed that the PLR was able to predict severe atherosclerosis with sensitivity of 61% and specificity of 59%.^1^

Previous research has also demonstrated that a high pre-procedural PLR is an independent predictor of no-reflow development in patients undergoing primary coronary angioplasty.^5^ The PLR could help to define persons with high-risk CAD who might need a more aggressive treatment approach and closer clinical follow-up, in an efficient, simple, and low-cost manner.
